# Carbapenem-resistant Enterobacterales (CRE) acquisition and molecular characterization following colistin monotherapy and colistin-meropenem combination therapy: findings from the AIDA randomized trial

**DOI:** 10.1186/s13756-025-01651-1

**Published:** 2025-11-05

**Authors:** Amir Nutman, Elizabeth Temkin, Jonathan Lellouche, Maayan Amar Ben Dalak, Ella Kaplan, Mor Lurie-Weinberger, Yael Dishon Benattar, Ami Neuberger, Anat Stern, Vered Daitch, Noa Eliakim-Raz, Emanuele Durante-Mangoni, Mariano Bernardo, Domenico Iossa, George Daikos, Anna Skiada, Ioannis Pavleas, Lena Friberg, Ursula Theuretzbacher, Leonard Leibovici, Mical Paul, Yehuda Carmeli, AIDA Study Group, Mical Paul, Yael Dishon Benattar, Yaakov Dickstein, Roni Bitterman, Hiba Zayyad, Fidi Koppel, Yael Zak-Doron, Sergey Altunin, Nizar Andria, Ami Neuberger, Anat Stern, Neta Petersiel, Marina Raines, Amir Karban, Leonard Leibovici, Dafna Yahav, Noa Eliakim-Raz, Oren Zusman, Michal Elbaz, Heyam Atamna, Vered Daitch, Tanya Babich, Yehuda Carmeli, Amir Nutman, Amos Adler, Inbar Levi, George Daikos, Anna Skiada, Ioannis Pavleas, Anastasia Antoniadou, Antigoni Kotsaki, Emanuele Durante-Mangoni, Roberto Andini, Domenico Iossa, Mariano Bernardo, Giusi Cavezza, Lorenzo Bertolino, Giuseppe Giuffre, Roberto Giurazza, Susanna Cuccurullo, Maria Galdo, Patrizia Murino, Adriano Cristinziano, Antonio Corcione, Rosa Zampino, Pia Clara Pafundi, Johan Mouton, Lena Friberg, Anders Kristoffersson, Ursula Theuretzbacher

**Affiliations:** 1 National Institute for Antibiotic Resistance and Infection Control, Tel Aviv Sourasky Medical Center, Tel Aviv, Israel; 2 Gray Faculty of Medical and Health Sciences, Tel Aviv University, Tel Aviv, Israel; 3 The Adelson School of Medicine, Ariel University, Ariel, Israel; 4 Institute of Infectious Diseases, Rambam Health Care Campus, Haifa, Israel; 5 The Ruth & Bruce Rappaport Faculty of Medicine, Technion – Israel Institute of Technology, Haifa, Israel; 6 Department of Medicine E, Beilinson Hospital, Rabin Medical Center, Petah Tikva, Israel; 7 AORN Dei Colli-Monaldi Hospital, Naples, Italy; 8 First Department of Medicine, Laikon General Hospital, and National and Kapodistrian University of Athens, Athens, Greece; 9 Intensive Care Unit, Laikon General Hospital, Athens, Greece; 10 Department of Pharmaceutical Biosciences, Uppsala University, Uppsala, Sweden; 11 Center for Anti-Infective Agents, Vienna, Austria; 12 University of Campania “Luigi Vanvitelli”, Naples, Italy; 13 The Cheryl Spencer Institute for Nursing Research, University of Haifa, Haifa, Israel; 14 Research Authority, Rabin Medical Center, Petah Tikva, Israel; 15 Wolfson Medical Center, Holon, Israel; 16 Rambam Health Care Campus, Haifa, Israel; 17 Beilinson Hospital, Petah Tikva, Israel; 18 Tel Aviv Sourasky Medical Center, Tel Aviv, Israel; 19 Laikon Hospital, Athens, Greece; 20 Attikon Hospital, Athens, Greece; 21 Monaldi Hospital, Naples, Italy; 22 Amsterdam, The Netherlands; 23 Uppsala University, Uppsala, Sweden; 24 Center for Anti-Infective Agents, Vienna, Austria

**Keywords:** Colistin, Meropenem, Combination treatment, Infection, Colonization, Carbapenem-resistant Enterobacterales (CRE), Carbapenemase-producing Enterobacterales (CPE), CRE acquisition, Antimicrobial resistance, Whole-genome sequencing

## Abstract

**Background:**

Colistin-carbapenem combination therapy is frequently used for carbapenem-resistant Gram-negative infections, but its impact on subsequent acquisition of carbapenem-resistant Enterobacterales (CRE) requires further investigation. We evaluated the incidence of CRE acquisition and performed molecular characterization of recovered isolates following treatment with colistin–meropenem versus colistin monotherapy.

**Methods:**

This analysis addressed a pre-specified secondary aim of the AIDA multicenter randomized controlled trial, which compared colistin monotherapy to colistin–meropenem combination therapy for carbapenem-resistant Gram-negative infections at six hospitals in Israel, Greece, and Italy. Rectal swabs were obtained at enrollment and weekly until day 28 or discharge. Swabs were processed centrally by plating onto MacConkey agar supplemented with imipenem to selectively isolate CRE. Recovered colonies were identified using MALDI-TOF mass spectrometry, and meropenem minimum inhibitory concentrations (MICs) were determined by broth microdilution. Clinical cultures were obtained as indicated and processed locally, and CRE isolates were sent to the central laboratory for confirmation and characterization. Whole-genome sequencing was used to determine sequence types and resistance genes. Patients were excluded if they had CRE detected at baseline, either by rectal culture or as the index clinical isolate, or if no follow-up rectal cultures were available.

**Results:**

Among 197 eligible patients (99 colistin; 98 colistin–meropenem), CRE acquisition occurred in 6 (3.0%): 1/99 (1.0%, 95% CI 0.03–5.5%) in the monotherapy arm and 5/98 (5.1%, 95% CI 1.7–11.5%) in the combination arm (*p* = 0.12). Two patients in the combination arm developed clinical infections caused by CRE (bacteremia and pneumonia); none occurred in the monotherapy arm. Carbapenemase genes were detected in four of the six acquired CRE isolates: one in the monotherapy arm (*bla*_VIM_) and three in the combination arm (all *bla*_KPC_). Identified species included *Klebsiella pneumoniae* and *Escherichia coli* belonging to established and emerging high-risk, multidrug-resistant clones.

**Conclusions:**

Patients treated with colistin-meropenem had a higher, though not statistically significant, rate of CRE acquisition. Early detection of high-risk CRE clones highlights the need to weigh potential unintended consequences when selecting combination regimens for multidrug-resistant infections.

**Trial Registration:**

AIDA trial was registered with ClinicalTrials.gov, number NCT01732250 (submitted 19-11-2012).

**Supplementary Information:**

The online version contains supplementary material available at 10.1186/s13756-025-01651-1.

## Background

Carbapenem-resistant Enterobacterales (CRE) represent a growing global health threat, associated with limited treatment options, delayed therapy, prolonged hospitalizations, and increased morbidity and mortality. These infections also impose a significant economic and operational burden on healthcare systems [1, 2].

In hospitalized patients, CRE acquisition typically results from a combination of endogenous and exogenous factors. Antibiotic-induced disruption of the gut microbiota can promote overgrowth of resistant organisms, while exogenous transmission may occur via contaminated surfaces, healthcare workers, or colonized patients [3]. Vulnerable populations—such as those with prolonged hospitalizations, invasive procedures, or immunosuppression—are particularly at risk, especially in settings with high colonization pressure and inadequate infection prevention measures [4, 5].

CRE colonization generally precedes infection [6], with rectal colonization serving as a reservoir for both clinical disease and onward transmission. A systematic review estimated that 16.5% of CRE-colonized patients go on to develop infection [7], although the risk varies by bacterial species and patient-related factors [8]. Preventing colonization is a key strategy to reduce CRE-related morbidity and depends, in part, on antibiotic selection, which influences both acquisition risk and resistance dynamics.

The rationale for combining polymyxins with carbapenems to treat infections caused by carbapenem-resistant organisms is supported by in vitro studies demonstrating synergistic activity between these antibiotic classes [9]. Synergism—where the combined effect exceeds the sum of individual effects—may enhance bacterial killing and also reduce the likelihood of resistance [9, 10]. However, translating this approach into clinical use requires caution: combination therapy increases overall antibiotic exposure and may inadvertently amplify selection pressure, fostering the spread of resistant organisms. Two recent randomized controlled trials, AIDA [11, 12] and OVERCOME [13], found no significant difference in clinical outcomes between colistin-meropenem combination therapy and colistin monotherapy for severe carbapenem-resistant infections.

These findings underscore the complexity of optimizing treatment strategies and highlight the need to assess potential unintended consequences—such as the acquisition of CRE. Here, we present the results of a pre-specified secondary aim of the AIDA trial: to assess the incidence of newly acquired CRE colonization or infection among trial participants, compare acquisition risk between those treated with colistin–meropenem versus colistin monotherapy, and characterize the molecular features of the acquired isolates.

## Methods

### Study design and participants

AIDA was conducted between October 2013 and December 2016 at six hospitals across Italy, Greece, and Israel. The trial enrolled inpatients aged 18 years and older who were diagnosed with infections caused by colistin-susceptible, carbapenem-non-susceptible Gram-negative bacteria. This analysis included participants from the AIDA trial whose index infection was not caused by CRE.

Rectal swabs for CRE detection were taken on the day of enrollment and weekly thereafter until day 28, discharge, or death. Clinical cultures were obtained as clinically indicated during routine care. Patients with a CRE-positive rectal culture at enrollment or lacking follow-up rectal cultures were excluded.

### Microbiological methods

Rectal swabs were inoculated in 5 ml of Brain Heart Infusion (BHI) enrichment broth (with manufacturers varying by site)**,** prepared according to the respective manufacturer’s instructions. After overnight incubation (approximately 18 h) at 35 ± 2 °C, an aliquot of 1 ml of broth was frozen at − 80 °C and shipped, along with the index isolate and any follow-up carbapenem-resistant clinical isolates, to a central reference laboratory in Tel Aviv, Israel, for processing.

Broths were thawed and 10 μl was inoculated on MacConkey agar supplemented with imipenem (1 μg/ml) (Hylabs, Rehovot, Israel). After overnight incubation at 35 ± 2 °C, suspicious colonies were identified by MALDI-TOF mass spectrometry (Vitek MS™, bioMérieux SA, Marcy-l'Étoile, France). For the colonies confirmed as *Enterobacterales*, meropenem minimal inhibitory concentration (MIC) was determined by broth microdilution. Multiplex-PCR was performed for detection of carbapenemase genes (*bla*_KPC_, *bla*_NDM_, *bla*_VIM_, *bla*_OXA-48_, *bla*_IMP_) [14].

CRE isolates were defined as *Enterobacterales* resistent to meropenem (MIC ≥ 4 μg/mL) or with a detectable carbapenemase gene, according to the U.S. Centers for Disease Control and Prevention (CDC) 2015 criteria [15]. If a carbapenemase gene was detected, the isolate was defined as carbapenemase-producing *Enterobacterales* (CPE); otherwise it was defined as non-carbapenemase-producing CRE (NCPCRE). Further MIC testing of CRE isolates, derived from either rectal or clinical cultures, against a panel of antibiotics was performed using GN4F and EURGNCOL Sensititre™ Plates (Thermo Fisher Scientific, Cleveland, OH, USA), according to the manufacturer's instructions. Briefly, an overnight culture was diluted to 0.5 McFarland, and then further diluted 1:1000 in Mueller–Hinton broth. Each well was inoculated with 50 μl of bacterial suspension and plates were sealed and incubated overnight at 35 ± 2 °C. Results were manually interpreted by visual inspection for bacterial growth. Antimicrobial susceptibility was interpreted according to Clinical and Laboratory Standards Institute (CLSI) guidelines [16]; for tigecycline, U.S. Food and Drug Administration (FDA) breakpoints were applied [17], as no CLSI criteria exist for *Enterobacterales*.

### Whole genome sequencing (WGS) and characterization

CRE isolates were sequenced using Illumina short reads (Illumina Inc., San Diego, CA, USA) at SNPsaurus (SNPsaurus LLC, Eugene, OR, USA). Sequence type (ST) was determined using pubMLST (https://pubmlst.org). Antibiotic resistance genes (ARGs) were detected using ResFinder version 4.6.0 (https://cge.food.dtu.dk/services/ResFinder/) and the Comprehensive Antibiotic Resistance Database (https://card.mcmaster.ca/home/).

### Statistical analysis

We used the Wilcoxon rank-sum test, chi-squared, or Fisher’s exact test to compare characteristics of patients in the two study arms (colistin monotherapy vs. colistin–meropenem combination therapy). CRE and CPE acquisition rates were calculated as the proportion of patients with either a follow-up rectal or clinical culture positive for CRE or CPE, with corresponding 95% confidence intervals (CIs). Acquisition rates in the two study arms were compared using Fisher’s exact test. Analyses were performed using SPSS version 29 (IBM Corp., Armonk, NY, USA).

## Results

**Fig. 1:**
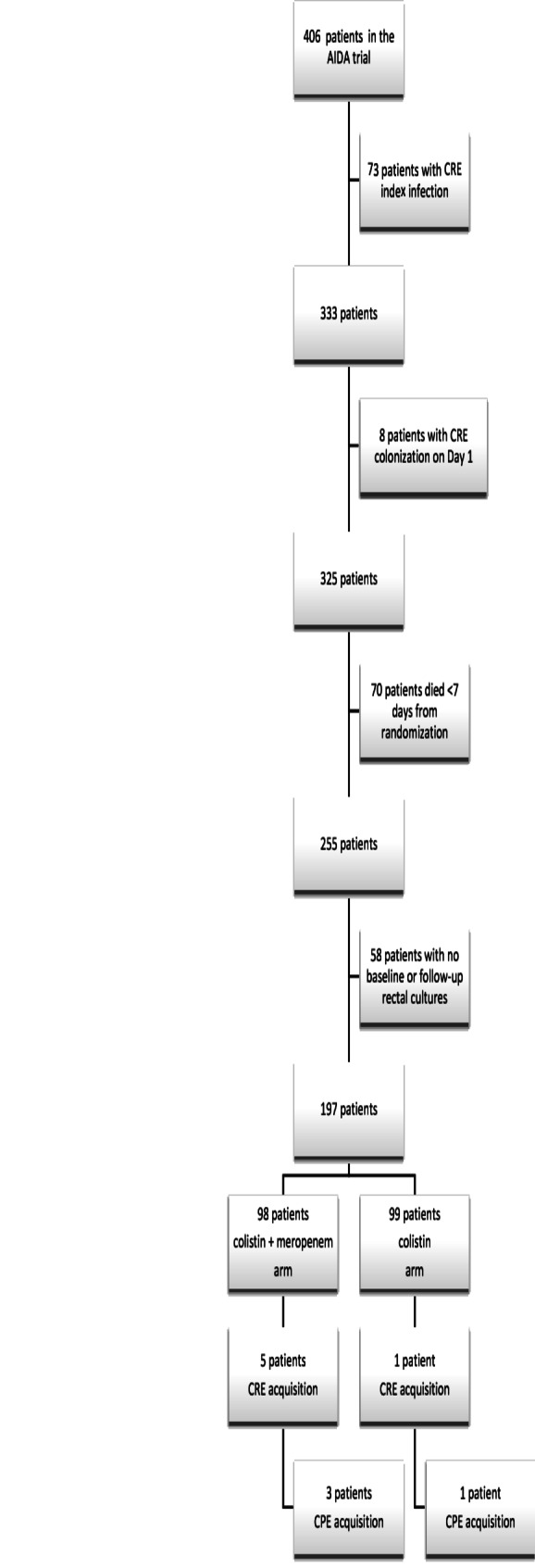
Flow chart of study inclusion. Abbreviations: CRE—Carbapenem-resistant *Enterobacterales*; CPE—carbapenemase-producing *Enterobacterales*

A total of 406 patients were enrolled in the AIDA trial. For this analysis, 73 patients with CRE index infections, 8 patients colonized at enrollment, 70 who died within 7 days of randomization, and 58 without baseline or follow-up rectal cultures were excluded, yielding a final sample of 197 patients (Fig. 1). Of these, 99 received colistin monotherapy and 98 received colistin–meropenem combination therapy.

Baseline characteristics were similar between groups (Table 1). There was no significant difference in 28-day mortality or days from randomization to death between groups (*p* = 0.5 for both comparisons).

**Table 1:** Characteristics of study participants, by treatment arm

		Colistin (N = 99)	Colistin and meropenem (N = 98)	*p-value*
*Demographic and medical characteristics*				
	Age (years), median (IQR)	66 (55–77)	67.5 (54–79)	0.86
	Female sex, no. (%)	40 (40.4)	39 (39.8)	1
	Country, no. (%)			0.11
	Greece	9 (9.1)	3 (3.1)	
	Italy	6 (6.1)	11 (11.2)	
	Israel	84 (84.8)	84 (85.7)	
	Admitted from, no. (%)			0.19
	Home	71 (71.7)	59 (60.2)	
	Long term care facility	7 (7.1)	16 (16.3)	
	Other hospital	21 (21.2)	23 (23.4)	
	BMI (kg/m^2^), median (IQR)	26 (23–31)	27 (24.6–29.6)	0.44
	Functional status, no. (%)			0.37
	Independent	6 (6.1)	2 (2)	
	Assistance in ADL	9 (9.1)	13 (13.3)	
	Bedridden	84 (84.8)	83 (84.7)	
	Charlson comorbidity index, median (IQR)	1 (0–3)	1 (0–3)	0.95
	Recent surgery, no. (%)	29 (29.3)	32 (32.7)	0.65
	Chronic renal failure, no. (%)	12 (12.1)	20 (20.4)	0.13
	Malignancy, no. (%)			0.43
	Solid	9 (9.1)	6 (6.1)	
	Hematological	3 (3)	1 (1)	
	Solid organ transplantation, no. (%)	3 (3)	5 (5.1)	0.5
	Bone marrow transplantation, no. (%)	1 (1)	0 (0)	1
	Immunosuppresive therapy, no. (%)	7 (7.1)	5 (5.1)	0.77
	Days from hospital admission to randomization, median (IQR)	16 (11–27)	17 (11.75–26)	0.77
*Status at infection onset (index culture taken time)*				
	Mechanical ventilation, no. (%)	77 (77.8)	72 (73.5)	0.51
	Hemodynamic support, no. (%)	17 (17.2)	15 (15.3)	0.85
	Hemodialysis, no. (%)	4 (4)	3 (3.1)	1
	Parenteral nutrition, no. (%)	11 (11.1)	7 (7.1)	0.46
	SOFA score, median (IQR)	5 (3–8)	5.5 (4–7)	0.9
	Arterial line, no. (%)	40 (40.4)	35 (35.7)	0.56
	Central venous catheter, no. (%)	47 (47.5)	53 (54.1)	0.39
	Urinary catheter, no. (%)	92 (92.9)	87 (88.8)	0.33
	Nasogastric tube, no. (%)	81 (81.8)	79 (80.6)	0.86
	Ventriculostomy, no. (%)	5 (5.1)	7 (7.1)	0.57
	Pacemaker, no. (%)	4 (4)	1 (1)	0.37
*Status at randomization*				
	Mechanical ventilation, no. (%)	75 (75.8)	74 (75.5)	1
	Hemodynamic support, no. (%)	14 (14.1)	17 (17.3)	0.56
	Hemodialysis, no. (%)	6 (6.1)	3 (3.1)	0.5
	SOFA score, median (IQR)	5 (3–7)	6 (4–8)	0.13
*Infection characteristics and treatment*				
	Place of acquisition of index infection, no. (%)			0.5
	ICU	40 (40.4)	34 (34.7)	
	Medical ward	49 (49.5)	47 (48)	
	Surgical ward	9 (9.1)	15 (15.3)	
	Community or other institution	1 (1)	2 (2)	
	Index pathogen, no. (%)			1
	* Acinetobacter baumannii*	95 (96)	94 (95.9)	
	* Pseudomonas aeruginosa*	3 (3)	3 (3.1)	
	Other	1 (1)	1 (1)	
	Type of infection, no. (%)			0.73
	Bacteremia	26 (26.3)	31 (31.6)	
	Pneumonia	67 (67.7)	59 (60.2)	
	Urinary tract infection	6 (6.1)	8 (8.2)	
	Modification of assigned regimen, no. (%)	10 (10.1)	5 (5.1)	0.19
*Outcome*				
	Died within 28 days, no. (%)	30 (30.3)	34 (34.7)	0.51
	Days from randomization to death, median (IQR)	14 (9–25.25)	18 (12–24)	0.51
	Acquired CRE, no. (%)	1 (1)	5 (5.1)	0.12
	Acquired CPE, no. (%)	1 (1)	3 (3)	0.37
	KPC	0	3	
	VIM	1	0	

IQR – interquartile range; BMI – body mass index; ADL – activities of daily living; SOFA—sequential organ failure assessment; ICU – intensive care unit; CRE – carbapenem-resistant *Enterobacterales*; CPE – carbapenemase-producing *Enterobacterales*

A total of six patients (6/197; 3.0%, 95% CI 1.1–6.5%) acquired CRE during follow-up: one in the colistin arm (1.0%, 95% CI 0.03–5.5%) and five in the colistin-meropenem arm (5.1%, 95% CI 1.7–11.5%); however, this difference was not statistically significant (*p* = 0.12). Two of the six patients developed clinical infections, both in the colistin-meropenem arm. CRE was identified by rectal screening in five patients and by a clinical culture in one patient, without a preceding rectal culture. These six cases originated from different study sites (Table 2). Among those with positive rectal cultures, CRE was detected on day 7 in four cases and on day 14 in one. In the patient without prior screening, CRE was isolated from a clinical specimen obtained on day 12. The single CRE isolate in the colistin arm was CPE (*bla*_VIM_); while three of the five isolates in the colistin-meropenem arm were CPE (all *bla*_KPC_). A comparison of patients who did and did not acquire CRE is presented in Supplementary Table S1.

**Table 2:** Characterization and MIC profile of acquired CRE isolates

Isolate No	Treatment Arm	Country	Species	Source	Days Post-Randomization	Ampicillin	Piperacillin	Ampicillin/ Sulbactam	Ticarcillin/ Clavulanate	Piperacillin/ Tazobactam	Cefazolin	Ceftriaxone	Ceftazidime/ Avibactam	Cefepime	Ceftolozane/ Tazobactam	Ertapenem	Imipenem	Meropenem	Doripenem	Aztreonam	Colistin	Amikacin	Tobramycin	Gentamicin	Tetracycline	Minocycline	Tigecycline	Levofloxacin	Ciprofloxacin
1	colistin	Greece	*K. pneumoniae*	Rectal screening	7	≥ 16 (R)	≥ 64 (R)	≥ 32/16 (R)	≥ 64 (R)	≥ 128/4 (R)	≥ 16 (R)	≥ 32 (R)	≥ 16/4 (R)	8 (I)	≥ 8/4 (R)	4 (R)	≥ 8 (R)	4 (R)	2 (I)	≥ 16 (R)	≥ 8 (R)	≥ 32 (R)	≥ 8 (R)	≥ 8 (R)	≥ 16 (R)	≥ 16 (R)	≤ 1 (S)	≥ 8 (R)	≥ 2 (R)
2	colistin-meropenem	Israel^	*E. coli*	Rectal screening	7	≥ 16 (R)	≥ 64 (R)	≥ 32/16 (R)	≥ 64 (R)	≥ 128/4 (R)	≥ 16 (R)	≥ 32 (R)	≤ 1/4 (S)	≥ 32 (R)	≥ 8/4 (R)	≥ 8 (R)	1 (S)	4 (R)	1 (S)	≥ 16 (R)	0.5 (I)	≥ 16 (R)	≥ 8 (R)	≥ 8 (R)	≥ 16 (R)	≤ 4 (S)	≤ 1 (S)	≥ 8 (R)	≥ 2 (R)
3	colistin-meropenem	Greece	*K. pneumoniae*	Rectal screening	14	≥ 16 (R)	≥ 64 (R)	≥ 32/16 (R)	≥ 64 (R)	≥ 128/4 (R)	≥ 16 (R)	≥ 32 (R)	≤ 1/4 (S)	≥ 32 (R)	≥ 8/4 (R)	≥ 8 (R)	≥ 8 (R)	≥ 16 (R)	≥ 4 (R)	≥ 16 (R)	4 (R)	≥ 32 (R)	≥ 8 (R)	≥ 8 (R)	≤ 4 (S)	8 (I)	≤ 1 (S)	≥ 8 (R)	≥ 2 (R)
4	colistin-meropenem	Israel^	*K. pneumoniae*	Rectal screening	7	≥ 16 (R)	≥ 64 (R)	≥ 32/16 (R)	≥ 64 (R)	≥ 128/4 (R)	≥ 16 (R)	≥ 32 (R)	4/4 (S)	≥ 32 (R)	≥ 8/4 (R)	≥ 8 (R)	≥ 8 (R)	≥ 16 (R)	≥ 4 (R)	≥ 16 (R)	0.5 (I)	≥ 16 (R)	≥ 8 (R)	≤ 2 (S)	≤ 4 (S)	8 (I)	≤ 1 (S)	≥ 8 (R)	≥ 2 (R)
5	colistin-meropenem	Italy	*K. pneumoniae*	Blood	12	≥ 16 (R)	≥ 64 (R)	≥ 32/16 (R)	≥ 64 (R)	≥ 128/4 (R)	≥ 16 (R)	≥ 32 (R)	≤ 1/4 (S)	16 (R)	≥ 8/4 (R)	4 (R)	4 (R)	2 (I)	2 (I)	≥ 16 (R)	≥ 8 (R)	≤ 4 (S)	≥ 8 (R)	≥ 8 (R)	≥ 16 (R)	8 (I)	≤ 1 (S)	≥ 8 (R)	≥ 2 (R)
6	colistin-meropenem	Israel^	*K. pneumoniae*	Rectal screening	7	Not available*																							

CRE – carbapenem-resistant *Enterobacterales*; MIC—Minimum Inhibitory Concentration; R- Resistant; I—Intermediate; S—Sensitive

^*^The isolate could not be successfully regrown from the stored specimen

^The three cases from Israel were from three different hospitals

Of the six acquired CRE isolates, five were further characterized; one organism could not be successfully regrown from the stored specimen. These included four *Klebsiella pneumoniae* strains belonging to ST258 (2 isolates), ST383, and ST392; and one *Escherichia coli* strain, ST8 (Table 3). Antimicrobial susceptibility testing revealed high levels of resistance to β-lactam antibiotics (Table 2). Colistin resistance was observed in 3/5 isolates (1/1 in colistin arm; 2/4 in colistin-meropenem arm), while 4/5 (80%) were susceptible to ceftazidime-avibactam. Non-β-lactam agents showed variable activity, with 100% susceptibility to tigecycline and 0% to tobramycin and fluoroquinolones.

**Table 3:** Genotypic characterization and antibiotic resistance genes of acquired CRE isolates

			Genes conferring resistance to:							
Isolate No	Species	MLST	Beta-lactames	Carbapenemes	Colistin	Aminoglycosides	Quinolones	Tetracyclines	Additional genes	OmpK37
1	*K. pneumoniae*	ST383	blaCMY-4, blaSHV-1, blaTEM-1, blaOXA-10	blaVIM-19	eptB, ArnT	rmtB, ant(2'')-Ia, aac(3)-IIa, aac(6')-Il, aph(6)-Id, aph(3'')-Ib, aadA24, aadA1, aadA2, aph(3')-Ia, ant(3'')-Ia	OqxA, OqxB	tet(G)	fosA, mph(A), floR, cmlA1, ARR-2, sul2, sul1, dfrA1	Yes
2	*E. coli*	ST8	blaCTX-M-15, blaTEM-1B, blaOXA-1	-	eptA, ArnT, PmrF	aac(3)-IId, aadA2b, aac(6')-Ib-cr, aadA1, ant(2'')-Ia	–	tet(A)	mph(A), catB3, cml, sul3, dfrA14, dfrA1	–
3	*K. pneumoniae*	ST258	blaSHV, blaTEM-84, blaOXA-9-like	blaKPC-2	eptB, ArnT	aadA2, aac(6')-Ib, aph(3')-Ia,	OqxA, OqxB	–	fosA6, mph(A), catA1, sul1, dfrA12	Yes
4	*K. pneumoniae*	ST258	blaSHV, blaTEM-84, blaOXA-9-like	blaKPC-3	eptB, ArnT	aac(6')-Ib	OqxA, OqxB	–	fosA6	Yes
5	*K. pneumoniae*	ST392	blaCTX-M-15, blaSHV-11, blaTEM-1A, blaOXA-1	blaKPC-3	eptB, ArnT	aph(6)-Id, aph(3'')-Ib, aac(3)-IIa, aac(6')-Ib-cr	OqxA, OqxB	tet(A)	fosA, catB3, qnrB1, sul2, dfrA14	Yes
6	*K. pneumoniae*	Not available*								

CRE – carbapenem-resistant *Enterobacterales*; MLST- Multilocus Sequence Typing

^*^The isolate could not be successfully regrown from the stored specimen

Resistance determinants identified by WGS aligned with antimicrobial susceptibility results (Table 3). All isolates carried at least two β-lactamase genes conferring resistance to penicillins and cephalosporins, as well as multiple aminoglycoside resistance genes. In addition, all strains harbored at least two *pmr*-mediated phosphoethanolamine transferase genes (*eptB*, *arnT*) associated with colistin resistance. Multiple efflux pump genes were identified in all isolates, and all *K. pneumoniae* strains encoded OmpK37, a porin associated with reduced antibiotic permeability.

## Discussion

This study found that 3.0% (6/197) of patients acquired CRE following treatment. Although the absolute number was low, the proportion was higher in the colistin–meropenem arm compared to the colistin monotherapy arm (5.1% vs. 1.0%, *p* = 0.12). This difference was not statistically significant, likely due to the rarity of the outcome and limited power to detect small differences.

Several studies have examined the relationship between antimicrobial exposure and the acquisition of carbapenem-resistant organisms. Hassoun-Kheir et al. [18] found that carbapenem exposure was significantly associated with acquisition of NCPCRE, but not with CPE, for which colonization pressure was the stronger predictor. Similarly, a case–control study from Singapore demonstrated higher odds of prior carbapenem exposure among patients with NCPCRE compared to CPE [19]. Additionally, a propensity-matched cohort study by Dallasheh et al. [20] found that patients treated with carbapenems had a higher likelihood of acquiring CRE compared to those treated with piperacillin-tazobactam. Bouganim et al. [5] reported that recent exposure to any antibiotic—not necessarily carbapenems—was a significant risk factor for NCPCRE acquisition.

Historically, NCPCRE have been described as arising de novo through porin loss or increased efflux in endogenous strains already carrying extended-spectrum β-lactamases (ESBLs) or AmpC β-lactamases, under antibiotic selective pressure [3, 21, 22]. In contrast, CPE are typically acquired from external sources via horizontal transmission of mobile carbapenemase genes [23, 24]. However, our findings suggest that both horizontal transmission and antibiotic selection pressure may have contributed to CRE acquisition. In 4/6 patients who acquired CRE in our study, CRE was detected as early as day 7, suggesting that colonization likely occurred during the initial hospitalization period. WGS identified globally disseminated, high-risk clones such as *K. pneumoniae* ST258, ST383, and ST392 [25, 26]. Notably, four of the six acquired CRE isolates were CPE, including three of five in the meropenem-treated arm. These observations, in the context of CRE-endemic hospital settings, raise the possibility that both CPE and NCPCRE strains were acquired through transmission events. The higher acquisition rate observed in the colistin–meropenem arm further suggests a potential role for antibiotic selection pressure, although this difference was not statistically significant.

It is biologically plausible that dual exposure to colistin and meropenem more profoundly disrupts the gut microbiota than monotherapy, diminishing colonization resistance and enabling CRE colonization. This is consistent with the known effects of broad-spectrum antibiotics, particularly meropenem, on the microbial ecosystem, which can create ecological niches favoring resistant organisms [27]. Infection control interventions such as contact precautions, active screening, patient cohorting, and dedicated staffing have proven effective in limiting transmission in endemic and outbreak settings [28]. Accordingly, preventing CRE acquisition requires a combined approach: minimizing unnecessary antibiotic exposure to reduce selective pressure and implementing robust infection control measures [29–31].

These findings highlight the importance of including colonization endpoints in antibiotic trials, as they offer critical insight into the unintended effects of treatment and inform stewardship and infection control strategies. This study, embedded in a randomized controlled trial with standardized protocols and prospective surveillance, illustrates that value. While this analysis included only a subset of trial participants, treatment groups were similar in baseline characteristics. However, the study was underpowered to detect modest differences in CRE acquisition. Detecting a four-fold difference would have required approximately 800 patients, while detecting a two-fold difference, over 4000.

Several limitations should be acknowledged. First, the small number of patients acquiring CRE precluded multivariable analysis to explore additional risk factors. Second, we lacked data on ward-level CRE colonization pressure. Nonetheless, the randomized design likely balanced this factor across treatment arms. Third, we did not assess patients’ gut microbiota composition or environmental contamination, limiting our ability to distinguish between endogenous overgrowth and exogenous transmission as mechanisms of CRE acquisition. Fourth, generalizability may be limited, particularly to settings with low CRE prevalence, as the study was conducted in endemic regions.

## Conclusions

Our findings raise the possibility that colistin–meropenem combination therapy increases the incidence of acquisition of CRE, including high-risk clones, particularly in endemic settings. While not statistically significant, the observed difference warrants further investigation. Amid the global rise in antimicrobial resistance, clinical trials offer a valuable opportunity to assess not only treatment efficacy but also potential downstream risks, such as antimicrobial resistance acquisition. Our findings underscore the importance of incorporating such outcomes to guide antibiotic stewardship and infection control strategies.

## Supplementary Information

Below is the link to the electronic supplementary material.

Supplementary Material 1

## Data Availability

Whole-genome sequencing data generated in this study have been deposited in the NCBI BioSample database under accession numbers SAMN48564268–SAMN48564273. All other data supporting the findings of this study are included in the manuscript and its supplementary material.

## Abbreviations

ARG: Antibiotic resistance genes
CPE: Carbapenemase-producing enterobacterales
CRE: Carbapenem-resistant enterobacterales
MIC: Minimum inhibitory concentration
NCPCRE: Non-carbapenemase-producing CRE
ST: Sequence type
WGS: Whole genome sequencing
