# Supplementary material for: Carbapenem-resistant Enterobacterales (CRE) acquisition and molecular characterization following colistin monotherapy and colistin-meropenem combination therapy: findings from the AIDA randomized trial
Source: Antimicrob Resist Infect Control. 2025 Nov 5;14:133. doi: 10.1186/s13756-025-01651-1 (PMC12590854; doi:10.1186/s13756-025-01651-1)
Supplement: Supplementary file 1 — Supplementary Material 1 [file 13756_2025_1651_MOESM1_ESM.docx]

**Table S1: Characteristics of study participants, by CRE acquisition (infection or colonization) status**

|  |  | **No CRE acquired (N=191)** | **CRE acquired (N=6)** |
| --- | --- | --- | --- |
| **Demographics and background** | | | |
|  | Age (years), median (IQR) | 67 (55-78) | 71 (23-82) |
|  | Female sex, no. (%) | 78 (40.8) | 1 (16.7) |
|  | Country, no. (%) |  | |
|  | Greece | 10 (5.2) | 2 (33.3) |
|  | Italy | 16 (8.4) | 1 (16.7) |
|  | Israel | 165 (86.4) | 3 (50.0) |
|  | Admitted from, no. (%) |  | |
|  | Home | 127 (66.5) | 3 (50.0) |
|  | Long term care facility | 23 (12.0) | 0 (0.0) |
|  | Other hospital | 41 (21.5) | 3 (50.0) |
|  | BMI (kg/m^2^), median (IQR) | 26.7 (24.0-30.8) | 28.1 (25.0-29.0) |
|  | Functional status, no. (%) |  | |
|  | Independent | 8 (4.2) | 0 (0.0) |
|  | Assistance in ADL | 21 (11.0) | 1 (16.7) |
|  | Bedridden | 162 (84.8) | 5 (83.3) |
|  | Charlson comorbidity index, median (IQR) | 1 (0-3) | 0 (0-1) |
|  | Recent surgery, no. (%) | 59 (30.9) | 2 (33.3) |
|  | Chronic renal failure, no. (%) | 31 (16.2) | 1 (16.7) |
|  | Malignancy, no. (%) |  | |
|  | Solid | 15 (7.9) | 0 (0.0) |
|  | Hematological | 4 (2.1) | 0 (0.0) |
|  | Solid organ transplantation, no. (%) | 8 (4.2) | 0 (0.0) |
|  | Bone marrow transplantation, no. (%) | 1 (0.5) | 0 (0.0) |
|  | Immunosuppresive therapy, no. (%) | 12 (6.3) | 0 (0.0) |
|  | Days from hospital admission to randomization, median (IQR) | 17 (11-27) | 10 (6-16) |
| **Status at infection onset (index culture taken time)** | | | |
|  | Mechanical ventilation, no. (%) | 145 (75.9) | 4 (66.7) |
|  | Hemodynamic support, no. (%) | 30 (15.7) | 2 (33.3) |
|  | Parenteral nutrition, no. (%) | 15 (7.9) | 3 (50.0) |
|  | Hemodialysis, no. (%) | 6 (3.1) | 1 (16.7) |
|  | SOFA score, median (IQR) | 5 (4-7) | 4.5 (2-11) |
|  | Arterial line, no. (%) | 72 (37.7) | 3 (50.0) |
|  | Central venous catheter, no. (%) | 97 (50.8) | 3 (50.0) |
|  | Urinary catheter, no. (%) | 173 (90.6) | 6 (100.0) |
|  | Nasogastric tube, no. (%) | 157 (82.2) | 3 (50.0) |
|  | Ventriculostomy, no. (%) | 12 (6.3) | 0 (0.0) |
|  | Pacemaker, no. (%) | 4 (2.1) | 1 (16.7) |
| **Status at randomization** | | | |
|  | Mechanical ventilation, no. (%) | 145 (75.9) | 4 (66.7) |
|  | Hemodynamic support, no. (%) | 29 (15.2) | 2 (33.3) |
|  | Hemodialysis, no. (%) | 8 (4.2) | 1 (16.7) |
|  | SOFA score, median (IQR) | 5 (4-7) | 4.5 (2-11) |
| **Infection characteristics and treatment** | | | |
|  | Place of acquisition of infection, no. (%) |  | |
|  | ICU | 70 (36.7) | 4 (66.7) |
|  | Medical ward | 94 (49.2) | 2 (33.3) |
|  | Surgical ward | 24 (12.6) | 0 (0.0) |
|  | Community or other institution | 3 (1.6) | 0 (0.0) |
|  | Index pathogen, no. (%) |  | |
|  | *Acinetobacter baumannii* | 183 (95.8) | 6 (100.0) |
|  | *Pseudomonas aeruginosa* | 6 (3.1) | 0 (0.0) |
|  | Other | 2 (1.1) | 0 (0.0) |
|  | Type of infection, no. (%) |  | |
|  | Bacteremia | 55 (28.8) | 2 (33.3) |
|  | Pneumonia | 123 (64.4) | 3 (50.0) |
|  | Urinary tract infection | 13 (6.8) | 1 (16.7) |
|  | Modification of assigned regimen, no. (%) | 15 (7.9) | 0 (0.0) |
| **Outcome** | | | |
|  | Died within 28 days, no. (%) | 63 (33.0) | 1 (16.7) |
|  | Days from randomization to death, median (IQR) | 17.5 (9-24) | 32 (23-37) |

Abbreviations: IQR – interquartile range; BMI – body mass index; ADL – activities of daily living; SOFA - sequential organ failure assessment; ICU – intensive care unit; CRE – carbapenem-resistant Enterobacterales
